# Prioritization of predisposing factors of gingival hyperplasia during orthodontic treatment: the role of amount of biofilm

**DOI:** 10.1186/s12903-021-01433-2

**Published:** 2021-02-24

**Authors:** Séverine Vincent-Bugnas, Leslie Borsa, Apolline Gruss, Laurence Lupi

**Affiliations:** 1 Département de parodontologie, Université Côte d’Azur, UFR Odontologie, 24 Avenue des diables bleus, 06300 Nice, France; 2 Pôle d’Odontologie, Centre Hospitalier Universitaire de Nice, 5 Rue Pierre Dévoluy, 06000 Nice, France; 3 Laboratoire MICORALIS EA7534, Université Côte d’Azur, 24 Avenue des diables bleus, 06300 Nice, France; 4 Département de santé publique, Université Côte d’Azur, UFR Odontologie, 24 Avenue des diables bleus, 06300 Nice, France; 5 Université Côte d’Azur, UFR Odontologie, 24 Avenue des diables bleus, 06300 Nice, France

**Keywords:** Gingival overgrowth, Orthodontic treatment, Risk factors, Dental plaque

## Abstract

**Background:**

The mechanism of gingival growth that may occur during fixed orthodontic treatment is not yet fully understood and the amount of dental plaque is often incriminated. The objective of this study was to evaluate the prevalence of gingival growth during multi-attachment orthodontic treatment and to prioritize its predicting factors, especially the quantity of biofilm.

**Methods:**

This comprehensive cross-sectional descriptive study was conducted on orthodontic patients aged 9 to 30 years, in good health, treated by a fixed appliance. Periodontal clinical parameters such as plaque index, gingival index, probing pocket depth, periodontal phenotype and gingival enhancement index were recorded. Likewise, the brushing habits and the date of the last scaling were noted. The orthodontic parameters studied were the duration of the treatment, the type of bracket, the alloys used for the arches and the type of ligatures. Descriptive statistics were carried out, and variables presenting *p* value < 0.25 were included in a multivariate analysis to calculate the Odds Ratio (OR) of gingival enlargement”.

**Results:**

A total of 193 patients were included (16.38 ± 4.89 years). Gingival growth occurred for 49.7% of patients included. The predisposing factors for this pathology during fixed orthodontic treatment were conventional metal brackets (*p* = 0.021), mouth breathing (*p* = 0.040), male gender (*p* = 0.035), thick periodontal phenotype (*p* = 0.043), elastomeric ligations (*p* = 0.007), duration of treatment (*p* = 0.022) and presence of plaque (*p* = 0.004). After achievement of the logistic regression, only two factors remained related to gingival enlargement: metallic brackets (OR: 3.5, 95% CI: 1.1–10.55) and duration of treatment (OR: 2.03, 95% CI: 1.01–4.08). The amount of plaque would not be directly related to the development of gingival increase during orthodontic treatment.

**Conclusions:**

Among the predisposing factors that underlie gingival growth during multi-attachment therapy, the amount of plaque is not found. The qualitative assessment of the plaque and its evolution during treatment could clarify the role of the biofilm in the occurrence of gingival overgrowth.

## Background

The effect of fixed orthodontic appliances on periodontal health has already been demonstrated [1–7]. Obviously, by hindering access to good oral hygiene and creating microbial shelters (both resulting in the accumulation of plaque), bonded orthodontic brackets impede good oral hygiene, resulting in a threat for periodontal health [7]. Among the periodontal diseases that occur during fixed orthodontic treatment, we find mainly orthodontic gingivitis or more precisely “gingivitis induced by bacterial biofilms and modified by local risk factors” in the new EFP classification [8], gingival recessions (or “peri-dental muco-gingival abnormalities”) and gingival growth [9]. Gingival enlargement (GE) is excessive growth of the gums where the inflammatory tissue may be in a limited region, or it may be generalized [1–4]. The three parts of gingival mucosa can be reached (marginal gingiva, interdental papilla and attached gingiva). It is most often due to an increase in the extracellular matrix of the mucosal chorion (collagen and fundamental substance with glycosaminoglycans such as hyaluronic acid, heparan sulfate or chondroitin sulfate, elastin, laminin or fibronectin) and more rarely bound to the epithelium.

The exact mechanism of this increase is not yet fully elucidated [9–11]; it is not necessarily associated with an increase in the number or size of fibroblasts. It is more likely a gingival enlargement or gingival increase “gingival overgrowth or gingival enlargement” than a strictly hypertrophy or gingival hyperplasia. Increased expression of type I collagen mRNA and regulation of growth of keratinocyte growth factor receptors may play an important role in excessive proliferation of epithelial cells and development of gingival growth [12].

The placement of orthodontic brackets leads to adverse changes in the composition of the bacterial plaque, both quantitatively and qualitatively, increasing both the periodontal risk and the carious risk. Indeed, an increase in *spirochetes, periodontal pathogens such as Prevotella Intermedia, Aggregatibacter actinomycetemcomittans*, *Porphyromonas gingivalis* or *Fusobacterium Nucleatum* [13] (as well as in *Candida* sp. [14]) is reported, and the presence of orthodontic brackets obviously makes the maintenance of good oral hygiene, particularly in interproximal spaces, much more difficult [15]. Finally, orthodontic treatments are often initialized during adolescence, that means when compliance and adherence to oral hygiene are quite difficult to obtain [16].

The accumulation of supra-gingival plaque then induces inflammatory alterations of the gingival tissues. However, the responses to this aggression both in their clinical form and in their time of appearance greatly vary with the individual [17]. These responses may depend on the quality and / or quantity of the biofilm and the host’s immune response to this aggression [18].

While some studies clearly indicate poor hygiene as responsible for gingival growth [19, 20], others demonstrate that gingival changes during orthodontic treatment are transient and do not imply any permanent alteration of periodontal tissue [4, 5, 21]. Although, these lesions, creating artificially deep periodontal pockets, must not be neglected and require treatment. Moreover, anterior GE promotes a negative impact on oral health-related quality of life of orthodontic patients [22].

To our knowledge, few studies have yet studied the predisposing factors associated with gingival growth during orthodontic treatment [1, 2]. The aim of this study was therefore to evaluate the prevalence of gingival growth during fixed orthodontic treatment and the factors related to it, using a hierarchical approach with a particular focus on the role of the amount of biofilm in its development.

## Methods

### Study population

All patients undergoing fixed orthodontic treatment were selected from patients treated in the dentofacial orthopedics department in the Nice University Hospital. The research council of the dental faculty of Nice University validated the study and the Delegation for Clinical Research and Innovation of University Hospital of Nice agreed to carry out this study. An informed written participant consent was obtained for each participant. For the minors included in the patient sample the consent to participate was obtained from a parent or guardian on behalf of the participants.

The study was a comprehensive cross-sectional survey: all patients, aged less than 30 years and treated with fixed attachments between october and november 2016 were eligible. Informed consent explaining the objectives of the study was signed and kept in the patients’ files.

Fixed corrective orthodontic treatment was carried out with conventional metal brackets, self-ligaturing brackets or ceramic brackets, straight wire technique, orthodontic arches fixed with simple elastomeric ligatures, metal ligatures, but without elastic chains, or proximal enamel stripping. Orthodontic rings (bands) were adapted to the molars with glass ionomer cement.

Patients suffering from congenital abnormality, systemic illness, cysts, or crevices, or with special needs or using systemic medication for the treatment of chronic diseases that might interfere with gingival overgrowth were excluded from the sample. Patients who required chemoprophylaxis before clinical examination were also excluded, as well as pregnant women and smokers.

The required number of subjects was estimated based on an expected difference of 10% with a theoretical proportion of 30% of GE [1]. Considering a power of 80% and a confidence interval of 95%, at least 172 persons were required.

We chose a face-to-face data collection, which provides a more accurate screening, with the operator’s (A.G) help.

Clinical examinations of all the patients were carried out by the same clinical operator (A.G.) previously calibrated by the members of the periodontal department. Training sessions were performed to ensure examiner reliability in regard to all the indices (Plaque Index, Gingival index, probing pocket depth and Seymour gingival growth index) [23] before the study started. Assessment of measurement reproducibility were conducted, for the Seymour gingival growth index only (other indices represent too variable conditions [1]), with 15 subjects. Replicate measurements were made by the examiner with a 2-day interval. An unweighted kappa value of 0.85 was obtained.

### Survey

A questionnaire was completed by the investigator for each patient. The survey developed for this study is provided as Additional file 1. The different items were collected and then checked using the patient’s file. Data entry was anonymous, and no information in the electronic file could be used to identify the source person. The form was structured in 3 parts:

- A first paragraph containing general data: The patients were interviewed using standardized modalities and a questionnaire with closed questions about sociodemographic data that included the following: age, gender, ethnicity, father’s or mother’s profession or patient’s own profession if appropriate, using the INSEE (French Institute for statistics and economic studies) Scale and then, for statistical purposes, grouped into 3 classes (management profession, employees, or without employment), medical status and drug treatments,
- The second paragraph dealt with periodontal health: the presence of overgrowth and, if any, the severity and localization were noted: The extent of gingival increment was classified as localized (< 4 papillae involved) or generalized (> 4 papillae involved) [24]. For the anterior segment, the degree of gingival thickening on both labial and lingual side of each five papillae was graded as follows: 0 = no increase, 1 = increase ≤ 2 mm, 2 = increase > 2 mm and the gingival encroachment was graded as: 0 = normal, 1 = papilla involving 1/3 of adjacent tooth crown half, 2 = papilla involving 2/3 of adjacent tooth crown half, 3 = papilla involving > 2/3 of adjacent tooth crown half. These two scores were added, thus giving the Seymour gingival growth index [23]. The proportion of subjects with a Seymour index value of 30 or greater based on the cutoff proposed by the index for the definition of clinically relevant GE [1, 23].

Evaluation of the quality of oral hygiene by Silness and Loë’s plaque index (PI) (0: absence of plaque; 1: plaque not visible to the naked eye but detachable with the probe, 2: plaque visible to the naked eye, 3: abundant plaque visible to the naked eye in the sulcus and on the marginal gingiva [25], gingival inflammation by the gingival index (GI) of Loë and Silness (0: absence of inflammation, 1: mild inflammation, 2: moderate inflammation and induced bleeding, 3: severe inflammation, spontaneous bleeding) [26] and gingival phenotype (thin: periodontal probe visible through the marginal gingiva, or thick: probe not visible by transparency) [27], the brushing habits (frequency, hygiene equipment), the date of the last periodontal scaling were also noted.

Breathing (nose or mouth breathing) is systematically collected in the patients’ file. It was assessed thanks to both visual assessment and two tests: lip seal test for 3 minutes and Glaze test (mirror’s test) [28].

The last paragraph concerned orthodontic data: treatment duration, type of brackets, alloys used for orthodontic arches, type of ligatures. All this information is systematically collected in the file and was just double checked by the examiner (A.G).

### Statistical analysis

The statistical data was collected in a spreadsheet and analyzed using IBM SPSS software version 25.0 (Statistical Package for Social Services, Chicago, IL, USA). Descriptive statistics were performed with flat sorting: frequencies for the qualitative variables. The influence of different variables on the presence of a gingival growth was studied. Some variables were “binarized” when the numbers were too small. The plaque index and the gingival index were dichotomized in a “yes/no visible plaque” and in a “yes/no gingival bleeding”. Age and duration of treatment, were changed into ordinary scales and treated as categorical variable in logistic regression analysis.

The enlargement was considered “present” if it concerned at least 4 papillae [24]. It was analyzed with Chi-square test for qualitative variables. The significance threshold was set at < 0.05. Potentially significant variables (*p* < 0.25) in univariate analyses were then entered into a multivariate logistic regression model and variables that remained significant were ordered to hierarchize associated factors. The forward stepwise likelihood ratio method for analyses was used, as well as the fitness index (Hosmer–Lemeshow test).

## Results

### Description of the sample

A total of 193 patients were included in the study. They were divided into 68 boys and 125 girls. The Caucasians were the most numerous (more than half) while the Maghrebians (or North Africans) accounted for about one third of our sample. All the patients were in good physical condition (Table 1).

**Table 1:** General characteristics of studied population

	Characteristics	%
Sex	Boys	35.2
	Girls	64.8
Age groups	9–13 years	7.3
	13–19 years	49.2
	20 years and more	43.5
Ethnicity	Caucasian	53.9
	From Maghreb	32.6
	From Africa	12.4
	From Asia	1.0
General health problems	None	86.5
	Related to oral sphere	6.2
	Not related to oral sphere	7.3

### Periodontal parameters

Considering that at least 4 papillae should be involved, gingival enlargements concerned nearly half of the patients treated with fixed attachments (49.7%) and boys were more affected (60.3%) than girls (44%) (*p* = 0.035). These gingival complications did not depend on ethnicity. No relationship could be found with general health (*p* = 0.41), whereas impaired ventilation had a significant impact on gum health: more than half of mouth breathers (53.6%) had gingival enlargement while only one-third (35%) of those who normally breathed, through the nose (*p* = 0.05) were concerned. The gingival phenotype was also found to be a predisposing factor for gingival growth since it was much more frequent when the periodontium was thick (61%) (*p* = 0.043).

Univariate analyses showed that the presence of bacterial plaque seems to have played a determining role in the occurrence of gingival hyperplasia since its prevalence increases gradually from 20% in the absence of plaque (IP = 0) to 72% in case of abundant plaque (IP = 3), passing through 46% if the plaque was detectable only after scraping with the probe (IP = 1) and 55% if it was visible to the naked eye (IP = 2) (*p* = 0.004). These results are different from those found by multivariate analysis which does not reveal the amount of dental plaque as predisposing factor in enlargement overgrowth.

However, brushing frequency did not appear to be statistically related to gingival enlargement (*p* = 0.89). As all the patients didn’t present periodontitis, the increase of periodontal probing highlights the presence of false pockets characteristic of gingival overgrowth related to orthodontic mechanics.

### Orthodontic parameters

Gingival overgrowth was more common with metal brackets (53%) than with ceramic brackets (26%) (*p* = 0.021). The presence of nickel in the arch did not influence their appearance (*p* = 0.18). On the other hand, elastomeric ligations appear to have clearly favored them (58%) compared to metal ones or self-ligating brackets (38.9%) (*p* = 0.007) (Tables 2, 3). Significant variables (sex, kind of ventilation, amount of plaque, gingival phenotype, type of ligations, type of brackets) in univariate analyzes, were therefore included in a logistic regression model. Thus, it became possible to hierarchize the predisposing factors for gingival overgrowth during orthodontic treatment with fixed appliance: conventional metal brackets increased the risk for GE by 3.5 (95% CI: 1.1–10.55) and the duration of treatment also increased the risk for GE by 2.02 (95% CI: 1.01–4.08). Significant predisposing factors in univariate analyses (gender, breathing, periodontal phenotype, plaque and ligations) became not significant in the logistic regression (Table 4).

**Table 2:** Orthodontic characteristics of studied population

	Characteristics	%
*Exclusive mouth breather*		
No	153	79.3
Yes	40	20.7
*Oral Hygiène (OHI) Plaque index*		
IP = 0: No plaque	24	12.4
IP = 1 Plaque visible after scratching at the probe	67	34.7
IP = 2 Plaque visible with naked eye	84	43.5
IP = 3 Abondance of plaque	18.0	9.3
*Frequency of brushing*		
At least twice a day	176	91.2
Once a day or less	17	8.8
*Gingival phenotype*		
Thick	59	30.6
Thin	134	69.4
*Type of orthodontic brackets*		
Metal	170	88.1
Ceramic	6	3.1
Maxillary ceramic and metal in the mandible	17	8.8
*Orthodontic arch type*		
Containing nickel	60	31.1
Steel	133	68.9
*Ligatures*		
Metal	7	3.6
Elastomerics	109	56.5
Both	72	37.3
Self-ligating	5	2.6

**Table 3:** Bivariate relationships between covariates and gingival status

Variables	Presence of gingival overgrowth (n)		*p*
	Yes	No	
*Sex *			
Girls	55	70	0.035
Boys	41	27	
*Age*			
9–13	11	3	0.18
13–19	56	39	
> 20	29	55	
* Ethnicity *			
Caucasian	50	54	0.56
Maghreb	34	29	
African	12	12	
Asian people	0	2	
* General health problem*			
None	81	86	0.41
Not related to oral health	8	4	
Related to oral health	8	6	
* Mouth breathing *			
Yes	82	71	0.04
No	14	26	
* Presence of plaque *			
None	5	19	0.004
Little	31	36	
Visible	47	37	
Large amount	13	5	
* Frequence of brushing *			
At least twice a day	87	89	0.89
Once a day or less	9	8	
* Periodontal phenotype*			
Thick	36	23	0.043
Thin	60	74	
* Type of bracket *			
Metal	90	80	0.021
Ceramic	6	17	
* Orthodontic arch*			
With nickel	26	35	0.18
Without nickel	70	62	
* Type of ligature *			
Elastomerics	63	45	0.0071
Metal or self-ligating	33	52	
* Duration of treatment*			
Less than 1 year	53	42	0.022
Between 1 and 2 years	47	20	
More than 2 years	25	6

**Table 4:** Final multivariate logistic regression model of occurrence of gingival enlargement

	OR adjusted	95% confidence intervals
Duration of treatment	0: less than 1 year	2.03 [1.01–4.08]*
	1: 1–2 years	
	2: more than 2 years	
Type of bracket	0: ceramic	3.5 [1.1–10.55]*
	1: metal	

*OR* odds ratio

## Discussion

In our study, considering that at least 4 papillae should be involved, according to the definition of “localized” form as described by Sibaud et al. [24], about half of the patients exhibited gingival overgrowth. Our study allows to hierarchize the predisposing factors for this side effect of fixed orthodontic treatment. Both the duration of treatment and the nature of the materials constituting orthodontic appliances appeared as predisposing factors. On the other hand, plaque, in its quantitative aspect (Plaque Index), does not appear directly related to the development of gingival growth.

Orthodontists tend to view the teenage period as very supportive of orthodontic treatment since usually all or most of the permanent teeth have erupted, while craniofacial growth can still be stimulated. This allows the teeth to be displaced and malocclusions corrected while maintaining favorable facial growth [29]. Thus, the majority of orthodontic treatments are undertaken during this period. However, in spite of the oral hygiene instructions systematically given at the beginning of treatment, young patients often have difficulties in maintaining a correct level of oral hygiene, especially during adolescence, where compliance is difficult to obtain [16] and hormonal changes may potentiate gingival inflammation [30]. However, in our study, while we would have expected to find more gingival overgrowth in girls for whom the hormonal impregnation is higher, boys were twice as often affected (*p* = 0.035). Androgenic factors may therefore be found histologically. Anyway, in the multivariate analysis, gender no longer appears to be a predisposing factor.

Inevitably, placement of a fixed orthodontic appliance creates plaque retention areas that make cleaning difficult [31]. However, the constant presence of bacterial plaque is inevitably accompanied by numerous side effects such as gingivitis, gingival overgrowth, demineralization of enamel (leading to precarious leucomas and even to caries) and, possibly in extreme cases, attachment loss [32, 33].

Adhesion of microorganisms to surfaces is a result of electrostatic interactions and Van-der-Walls forces [34]. Although it is clear that initial attachment is an important factor governing colonization, the principle of adhesion and subsequent growth of the biofilm may differ significantly [35]. Once initial adhesion is obtained, other factors may influence further colonization. Reduced wettability can inhibit direct adhesion and thus plaque formation on orthodontic devices [36]. Thus, the nature and the surface characteristics of both orthodontic brackets and composite may influence the retention of the biofilm. The method of ligation of the archwire is an additional factor of importance for plaque retention. Some studies have investigated the effects of fixed orthodontic appliances on the microbial flora profile, and, among them, few have compared the effects of bracket architecture—specifically. Metallic orthodontic brackets have already been found to induce specific changes in the buccal environment such as decreased pH, increased accumulation and elevated S. mutans colonization [37, 38].

Besides, the archwire ligation method—induces a quantitative evaluation of the bacterial accumulation that occurs with the bonding of fixed appliances [7, 39–41]. In our study, the most common method of arch-wire ligation, elastomeric ties, was chosen as the basis of comparison against the SL mechanism and steel ligatures. They confirmed the propension for elastomeric ligations to retain plaque. However, this predisposing factor disappeared in the logistic regression.

In the same way, periodontal health is known to be a crucial element to consider before starting orthodontic treatment. If orthodontists are very much used to being cautious in case of proven periodontal disease or reduced periodontium after healing of a past pathology, the gingival phenotype must also be taken into consideration. The importance of the amount of keratinized tissue to maintain periodontal health has long been debated. Some have shown that the presence of keratinized tissue was not essential for periodontal health in the absence of plaque [42], or that the periodontitis could remain healthy even in the case of low height (at least 2 mm) and attached gingiva thickness combined with control of plaque control [43]. However, these reduced narrow and thin gingival conditions represent well and truly a risk factor for the development of gingival recessions, especially if they are associated with a short vestibule, trauma or poor hygiene. Recently, studies with a long-term clinical follow-up (between 18 and 35 years) have shown that the transformation into a thick gingival phenotype by an epithelio-conjunctive graft favors the health of periodontal tissues [44]. A thick periodontium, in response to bacterial aggression, will tend to thicken and form periodontal pockets. Thus, in our study, hyperplasia was much more common in the case of thick periodontium (61%) than in the case of thin periodontium (44.8%) (*p* = 0.043), but once again this predisposing factor disappeared in the logistic regression.

In our study, although the amount of plaque seemed to have played a determining role in the occurrence of gingival overgrowth in univariate analyses, since its prevalence increased gradually following a real gradient (*p* = 0.004), the role of the dental plaque quantity, after completion of the logistic regression, as apprehended by the index of Silness and Löe, seems to fade in favor of the other predisposing factors. This interpretation is confirmed by the fact that the frequency of brushing did not appear to be statistically related to gingival enlargement (*p* = 0.89). The explanation could lie in the qualitative and non-quantitative evolution of the dental plaque during fixed orthodontic treatment. This could lead to the qualitative selection of pathogens and the interindividual differences that might explain the different patterns of response and time needed for evident clinical responses, as well as local and systemic individual resistance or even a specific microbial challenge [17, 21, 45]. This finding seems to be supported by several microbiological studies which demonstrate that when fixed orthodontic appliances are placed, the potential for qualitative [46, 47] changes in the microbial composition of these areas enhances. Thus, periodontal reaction might be elicited by a change in the composition of the microbiological environment [21], even if the amount of plaque is not significantly involved.

So, gingival overgrowth was more frequent with metal brackets (53%) than with the ceramic ones (26%) (*p* = 0.021), the presence of nickel in the arches did not influence their appearance (*p* = 0.18), and elastomeric ligatures appear to have clearly favored them (58%) compared with metal ligatures or self-ligating brackets (38.9%) (*p* = 0.007) (Tables 2, 3).

The strengths of our study lie in supplying data on a commonly met condition during fixed orthodontic treatment (gingival enlargement). The originality comes from the ranking of predisposing factors.

The limitations of our study arise from the cross-sectional design, which only provides a snapshot of our outcome at a specific point in time and provides no indication of the sequence of events. Besides, we chose to study specific conditions that could appear to be potential predisposing factors for GE, but we might have omitted other ones that could have been of interest.

This study could contribute to improve clinical practice since awareness of related factors can guide the choice of practitioners or help them to advise their patients.

## Conclusions

Our initial hypothesis, namely the impact of the amount of biofilm in the occurrence of GE has not been verified. After completion of the logistic regression, the quantity of plaque would not be directly related to the development of this gingival increase. On the contrary, two conditions appeared to be real predisposing factors: the constituent material of the bracket and the duration of treatment. Thus, these results raise a new hypothesis: the quality of biofilm, instead of the amount of plaque, could be at the origin of the development of GE during orthodontic treatment. Therefore, studies on the qualitative evolution of plaque during fixed orthodontic treatment are needed to clarify the role of biofilm in the occurrence of gingival overgrowth.

## Supplementary Information

**Additional file 1.** Use of data for research purposes.

## Data Availability

The datasets used and/or analyzed during the current study are available from the corresponding author on reasonable request.

## Abbreviations

GE: Gingival enlargement
GI: Gingival index
OR: Odds ratio
PI: Plaque index
PPD: Pocket probing depth
