# Supplementary material for: Prioritization of predisposing factors of gingival hyperplasia during orthodontic treatment: the role of amount of biofilm
Source: BMC Oral Health. 2021 Feb 24;21:84. doi: 10.1186/s12903-021-01433-2 (PMC7903590; doi:10.1186/s12903-021-01433-2)
Supplement: Supplementary file 1 — Additional file 1. Use of data for research purposes. [file 12903_2021_1433_MOESM1_ESM.docx]

Use of data for research purposes

For patients and their parents

Sir, Madam,

Thank you for reading this information carefully;

You (your child) are (is) ongoing an fixed orthodontic treatment. As part of your (his/her) care at the University Hospital Center of the CHU de Nice (CHU de Nice), clinical data is collected in your (his/her) medical file.

The collected information is likely to be computer processed electronically for research purposes.

This research, placed under the responsibility of Prof. Lupi from the Odontology Department and of the Micoralis laboratory, aims to establish the relationships between gingival hyperplasia and fixed orthodontic treatment.

In accordance with the law "Informatique et Libertés" of January 6th, 1978 amended in 2004, you have a right to access and rectification to your personal data (or your child’s), by contacting the Pole Odontologie, 5 rue Pierre Dévoluy 06000 NICE.

You can also, for legitimate reasons, oppose the processing of your (his/her) data.

For any other questions, your orthodontist in the Nice Hospital, (Tel: 04.92.03.77.77), can provide you with the necessary information.

**To be filled in by the patient**

Last name: First name:

I object to the use of my clinical data for research purposes

I do not object to the use of my clinical data for research purposes

Date:

Signature:

Questionnaire

Patient

Age

Sex : Male Female

Ethnic Group :

Socio-Professional Category

|  | Supervisory staff | Field staff | other |
| --- | --- | --- | --- |
| father |  |  |  |
| mother |  |  |  |
| patient |  |  |  |

Health condition/ health status:

Medical treatment :

Oral ventilation : Presence Absence

**Periodontal examination :**

date of last descaling : < 6 months < 1 year > 1 year > 5 years or never

toothbrushing frequency : less than once a day once a day twice a day or more

Silness and Löe plaque index (1964): 0 1 2 3

Gingival biotype (Maynard and Wilson, 1981): Thin Thick

Presence of gingival growth : Yes No

History of periodontal surgery: Yes No

**Fixed orthodontic treatment :**

Duration (in semesters):

Brackets : metal ceramic both

Orthodontic wire : Niti round Niti rectangular

Stainless steel round Stainless steel rectangular TMA

Method of ligation : elastic stainless steel both power chain
